# The Screening Tomosynthesis Trial with Advanced Reader Methods (STREAM): design and rationale of a population-based breast cancer screening trial

**DOI:** 10.1007/s00330-024-11324-z

**Published:** 2025-01-09

**Authors:** Lindy Kregting, Daan van den Oever, Lian Pennings, Ruud Pijnappel, Nicolien van Ravesteyn, Ellen Verschuur, Marja van Oirsouw, Loes Dunning, Hans ‘t Mannetje, Ruben van Engen, Adriana Bluekens, Maartje Smid-Geirnaerdt, Cary van Landsveld-Verhoeven, Nehmat Houssami, Ioannis Sechopoulos, Mireille Broeders

**Affiliations:** 1 Radboud University Medical Center, IQ Health science department, Nijmegen, The Netherlands; 2 Radboud University Medical Center, Department of Medical Imaging, Nijmegen, The Netherlands; 3 Dutch Expert Centre for Screening (LRCB), Nijmegen, The Netherlands; 4 Erasmus MC, University Medical Center Rotterdam, Department of Public Health, Rotterdam, The Netherlands; 5 Dutch Breast Cancer Association, Utrecht, The Netherlands; 6 The Dutch Organisation for Population Screening, Utrecht, The Netherlands; 7 Elisabeth-Tweesteden Hospital, Department of Radiology, Tilburg, The Netherlands; 8 The Daffodil Centre, the University of Sydney—Joint venture with Cancer Council NSW —Faculty of Medicine and Health, Sydney, Australia; 9 University of Twente, Multi-Modality Medical Imaging, Enschede, The Netherlands

**Keywords:** Mammography, Breast neoplasms, Mass Screening, Cost-Benefit Analysis, Early Detection of Cancer

## Abstract

**Objectives:**

It is uncertain what the effects of introducing digital breast tomosynthesis (DBT) in the Dutch breast cancer screening programme would be on detection, recall, and interval cancers (ICs), while reading times are expected to increase. Therefore, an investigation into the efficiency and cost-effectiveness of DBT screening while optimising reading is required.

**Materials and methods:**

The Screening Tomosynthesis trial with advanced REAding Methods (STREAM) aims to include 17,275 women (age 50–72 years) eligible for breast cancer screening in the Netherlands for two biennial DBT screening rounds to determine the short-, medium-, and long-term effects and acceptability of DBT screening and identify an optimised strategy for reading DBT. The control group will consist of 86,400 women selected from the database of the Dutch breast cancer screening programme screened with digital mammography. The intervention group will undergo DBT examinations only. Four different reading strategies will be evaluated on a subset of first-round screening exams. These four strategies will also be evaluated combined with replacing one of the two readers with AI predictions. The Microsimulation Screening Analysis (MISCAN)-Breast model will be used to estimate the long-term outcomes of DBT screening assuming the best-performing reading method.

**Results:**

The primary outcome measure is the IC and advanced cancer rate at the second round (combined endpoint) in the DBT group compared to the control group. Secondary outcome measures are participation, recall and detection rates, positive predictive value, acceptability, reading method with the best case-based area under the curve and reading time, predicted breast cancer mortality, number of cancers overdiagnosed, and cost-effectiveness.

**Key Points:**

***Question*** *The short-, medium-, and long-term effects of digital breast tomosynthesis (DBT) imaging in the Dutch breast cancer screening programme are unknown, but essential to decide about implementation.*

***Findings*** *This protocol paper describes the primary endpoint of the STREAM trial: the combined interval and advanced cancer detection rate at the second DBT round.*

***Clinical relevance*** *The STREAM trial is a prospective, non-randomised, population-based study in the Dutch breast cancer screening programme, that aims to evaluate the effects and acceptability of two rounds of DBT screening to determine if DBT can enhance the programme’s outcomes.*

**Graphical Abstract:**

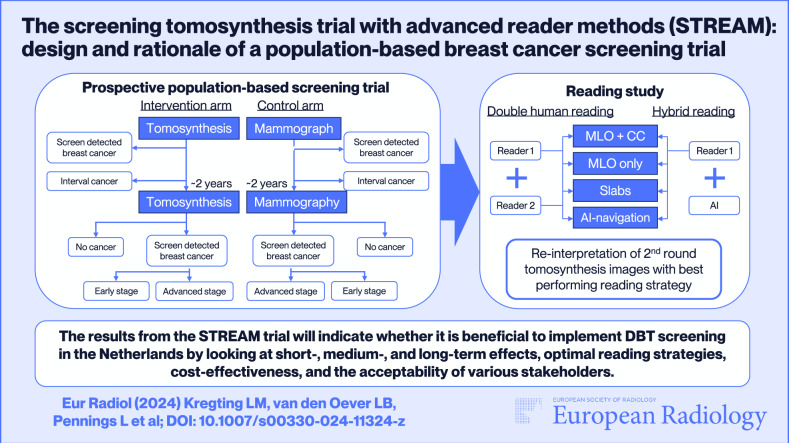

## Introduction

The Dutch breast cancer screening programme, hereafter the programme, offers a biennial digital mammography (DM) examination to asymptomatic women aged 50 to 75. Craniocaudal (CC) and mediolateral-oblique (MLO) examinations are double-read by two screening radiologists independently and women with suspicious findings are referred to a hospital for diagnostic work-up [1].

In 2019, the detection and recall rates of the programme were 6.9 per 1000 women screened and 2.4%, which are among the highest and lowest, respectively, in Europe [2, 3]. Due to workforce issues, the screening interval has increased over the last years (towards 32 months), thereby increasing the detection rate to 7.9 per 1000 in 2022 at the same recall rate [4].

However, the 2D nature of DM results in well-known limitations [5]. Digital breast tomosynthesis (DBT) is an imaging technique that has been developed as a potential alternative to DM. Multiple studies have concluded that DBT improved screen-detected breast cancer rates, but that no effect has been seen on interval cancer (IC) rates [6, 7]. However, it should be noted that only two studies with DBT plus synthetic mammography (SM) reported IC rates [7]. Houssami et al also emphasised that their results were derived from studies on initial DBT examinations and that results on subsequent examinations may be different.

On average, DBT screening in European trials was found to increase the cancer detection rate to an average of 7.2 per 1000 for biennial screening [7]. Since the Dutch programme already nearly reaches this detection rate with biennial DM, it is unclear if DBT would increase detection. In addition, in Europe, recall rates for DBT screening decreased or increased, depending on the baseline DM recall rate [8]. Therefore, the already-low recall rate in the Netherlands could be expected to increase with DBT screening, but it is unknown to what extent. It is important to investigate the effects and acceptability of DBT in the Dutch screening setting to predict if DBT would be cost-effective [9, 10].

For DBT screening in the Netherlands to be feasible from an economic and human resources point of view, the interpretation of DBT cases must be optimised, to result in an acceptable reading time [11]. Options available for reducing reading time include using the MLO view without the CC view [5], 6 mm slabs instead of 1 mm slices [12], or using AI-aided navigation [13]. In addition, replacing one of the readers with AI could also be an option to further reduce the radiologist’s workload [14]. Potentially, one or a combination of these approaches could reduce the DBT reading demands to even below those currently required in DM screening.

Therefore, the aim of the Screening Tomosynthesis trial with advanced REAding Methods (STREAM) is to determine the short-, medium-, and long-term outcomes and cost-effectiveness of nationwide DBT breast cancer screening in the Netherlands, when an optimal image acquisition and interpretation strategy is used, and to ensure its acceptability by the various stakeholders. Short-term indicators for the study are participation rate, prevalent recall rate, detection rate, and positive predictive value of DBT screening. Medium-term indicators comprise the advanced cancers (ACs) (stage ≥ II) detected in the second round of DBT screening, IC rate, and incident recall rate. Long-term indicators are predicted breast cancer mortality, number of cancers overdiagnosed, and cost-effectiveness.

## Methods and analysis

The Minister of Health, Welfare and Sport has granted ethical approval and a license to conduct this trial from 1 May 2023 to 1 June 2028 (Registration number: 3574709-1044141-PG). This is considered equivalent to approval by an institutional review board.

### Study design

**Fig. 1:**
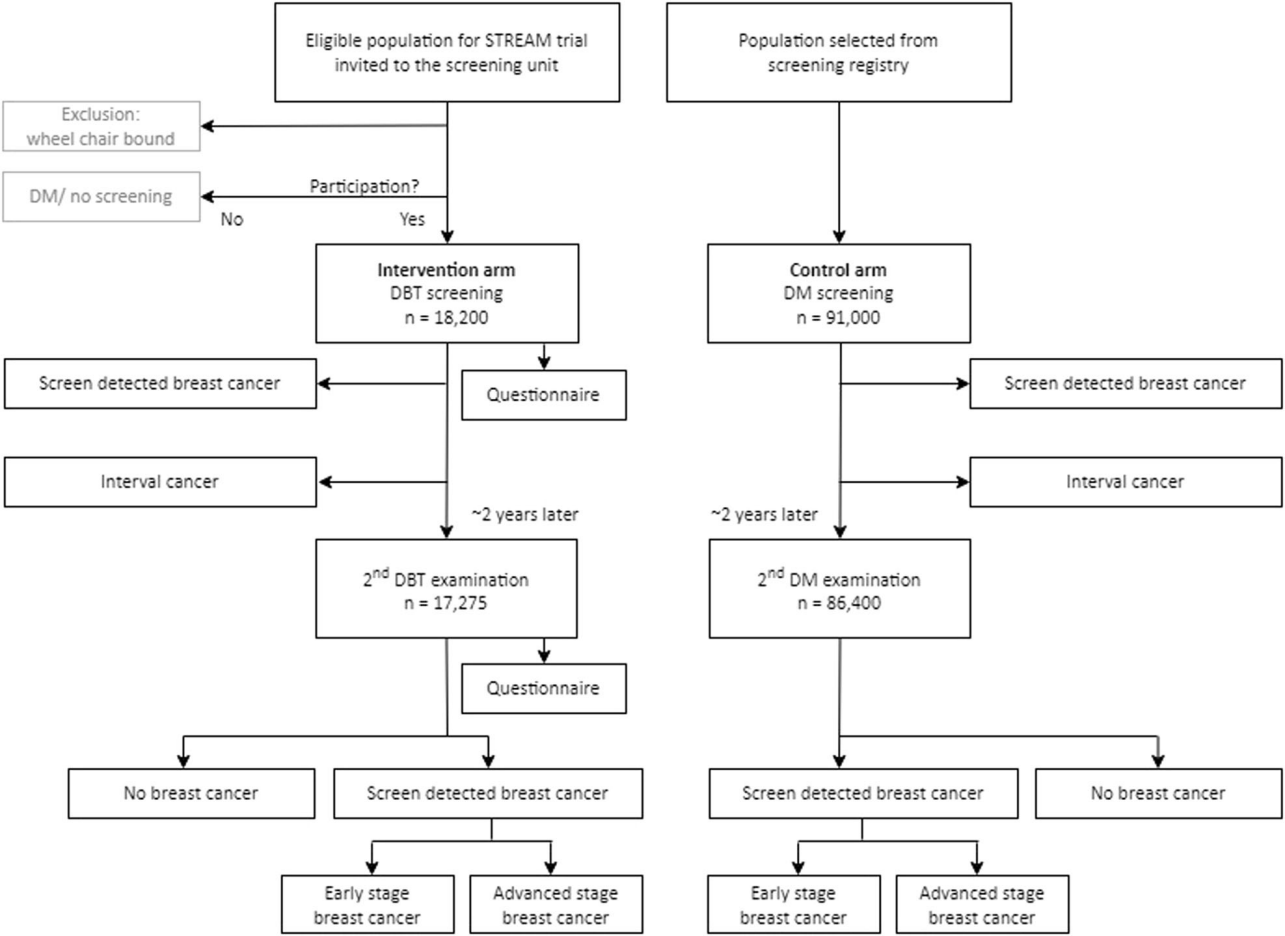
Flowchart of the recruitment and data collection in the two screening rounds of the STREAM trial

STREAM is a prospective, non-randomised, parallel-group population-based trial embedded in the Dutch programme. The intervention arm will consist of 17,275 participants undergoing two rounds of DBT imaging and the control group will consist of 86,400 participants undergoing two rounds of DM screening acquired around the same time as the DBT examinations (Fig. 1).

### Data collection

#### Population and recruitment strategy

In collaboration with the Dutch Cancer Screening Organisation (BVO-NL, due to its name in Dutch), three mobile and three fixed screening units throughout the Netherlands were selected to perform DBT screening based on geographical spread, availability, and capacity. All women between the ages of 49 and 72 who are due for screening in one of these units will receive an invitation to participate in the intervention arm of the STREAM trial. Invitations are sent with the invitation for screening and include an information leaflet and informed consent form. Women who decide to participate in the trial must provide written informed consent. Inclusion of participants started in July 2023 and was completed in May 2024. The only exclusion criterion is being wheelchair-bound, because of practical reasons. Participants will be re-invited for a second round of DBT screening, approximately two years later. See Fig. 1.

The control arm will be selected to be representative of the population of the intervention arm, with respect to age, region, and screening interval, but screened at different units. For the control arm, aggregated data will be received on the same parameters as the intervention group including follow-up. In the Netherlands, informed consent is implied for the use of pseudonymised or aggregated cancer screening data when one takes up the offer of screening unless the individual specifically opts out. Individuals who withdrew consent will not be included in the control arm.

#### Training

All radiographers and screening radiologists involved in the STREAM trial were trained before the start of recruitment. A one-day training was organised at the Dutch Expert Centre for Screening (LRCB) for the screening radiologists. The radiographers attended an online information meeting organised by BVO-NL and LRCB and were subsequently trained by BVO-NL and the application specialist of the system vendor.

#### Image acquisition

At the screening unit, the participants undergo two-view DBT imaging (i.e., CC and MLO views) of both breasts using the imaging systems currently in use by the programme (Selenia 3Dimensions, Hologic Inc.).

#### Image interpretation

In addition to the reconstruction of the DBT images from the acquired projections, the acquisition systems generate the corresponding SM images for the four views, all of which are made available to the interpreting radiologist. If available, DM images of previous screening examinations are used for comparison. DBT images are independently double-read, just like DM images are in the regular programme [15]. Discrepancies between the two radiologists are solved by arbitration or consensus, also as per current practice.

#### Acceptability for participants

After the DBT examination, participants are asked to complete either a web- or paper-based (according to their preference) questionnaire about the acceptability of DBT screening. Questions include topics on the experience of undergoing DBT screening, comparison to previous experience with DM, if any, intention to participate in subsequent DBT screening, suggestions for improvement, perceived pain or discomfort, and anxiety experienced during the DBT examination [16] (see Supplementary Appendix A). After the second round of DBT screening, participants will be asked to complete another comparable questionnaire.

In addition, radiographers and radiologists involved in the trial are asked to complete a questionnaire after the first and second rounds of DBT examinations. These questionnaires include questions about workflow, acquisition, perceived reading time, preferences, and suggestions for improvement (see Supplementary Appendix B).

A plan of action will be made to overcome the identified barriers based on the answers given in the questionnaires after the first round of DBT screening by participants, radiographers, and radiologists. When possible, improvements will be made for the second round of DBT screening. Questions about the effectiveness of the improvements will be added to the questionnaires after the second round of DBT screening.

#### Sample size calculations

Sample size calculations were done in G*Power (version 3.1.9.4. using an alpha of 0.05, a beta of 0.20, two-sided *Z*-test and an allocation ratio for the control/intervention group of 5) based on the primary outcome measure; i.e., the sum of the IC and advanced cancer (AC) rate at the seco nd round (combined endpoint) in the DBT group compared to the control group. We powered the trial to detect a reduction in the primary outcome measure of 1.3 per 1000, which reflects a difference of 36% from this combined endpoint with DM (3.6 per 1000) (see further details in Supplementary Appendix C). This requires a sample size for the intervention group in the second round of 17,275 screening examinations with DBT. Assuming 95% re-attendance, this requires 18,184 screening examinations with DBT in the first round. The sample size for the control group is five times that of the intervention group in the second round, i.e., 86,375 participants screened with DM. Again, assuming a 95% re-attendance rate, 90,693 participants are required. For practical reasons, the sample sizes for the first round were rounded off to 18,200 in the intervention group and 91,000 in the control group.

## Data analysis

### Statistical analyses

The IC rate will be defined as the number of cancers, both in situ and invasive, diagnosed clinically (i.e., outside of screening) after a negative DBT in the first, second, and third year after the first screening round of the trial per 1000 screens (considering the longer screening interval). The AC rate will be defined as the number of tumours stage II or higher (TNM classification [17]) diagnosed after recall in the second screening round of the trial per 1000 screens. Data on screen-detected cancers (SDC) and IC, as well as their characteristics (e.g., stage and grade), will be obtained through linkage with the Netherlands Cancer Registry and the Dutch nationwide pathology databank for the intervention as well as the control group.

Secondary outcome measures including participation rate (i.e., percentage of participants in DBT or DM screening of the population invited), recall rate (i.e., number of women recalled for further examination per 1000 women screened), detection rate (i.e., number of women with SDC per 1000 women screened), and positive predictive value (i.e., percentage of women with SDC of women recalled) will be calculated for both screening rounds separately. The recall rate in the first round is expected to be higher due to the first-round effect of a more sensitive screening test, the learning effect for the radiologists, and the absence of prior DBT screens to compare to [18, 19]. The recall rate of the second round will estimate the long-term recall rate for DBT screening. The detection rate will be calculated for invasive and in situ cancers, overall and for cancer stage II or higher. Screening outcomes of both rounds will be stratified by breast density, as assessed by automated methods by Volpara (Mātakina Technology) and Quantra (Hologic).

Screening outcomes for the two groups will be compared using the *χ*^2^ test to test differences between proportions and rates, overall and stratified by screening interval and breast density. The Fisher exact test will be used in stratified analysis with small groups. 95% confidence intervals will be calculated to consider the variability of proportions and rates. In addition, logistic regression analysis will be used to compare the results in the intervention and the control group by calculating the relative rates of the main screening detection measures, with 95% confidence intervals. The pain (scale 0–10), discomfort, and anxiety scores (4-point Likert scale) experienced in DBT screening will be compared to the experience of previous DM screening with paired *t*-tests and to scores on DM screening reported by a reference population with independent *t*-tests. A *p*-value ≤ 0.05 will be considered to indicate a statistically significant difference. Qualitative analyses in ATLAS.ti will investigate the answers to open questions on acceptability among participants, radiologists, and radiographers using inductive reasoning.

### Reading strategies

Multiple commercially available AI software solutions will be evaluated using all the data from the first round of the intervention arm. The software aims to detect malignancies in DBT images, highlight their location, and give a case-based and lesion-based score of probabilities of breast cancer. In agreement with the software vendors, the software description and the results will be reported anonymously. First, case-based probability of malignancy will be used to build recall rate/cancer detection rate curves and calculate the area under the receiver operating characteristics curve (AUC). All AI solutions with an AUC within 0.05 of the best-performing system will be used for the subsequent observer study.

With it or them, we will perform reader study sessions with ten screening radiologists to compare reading times with a dataset of 200 examinations. The dataset will be assembled from first-round cases and will include both cancer and negative cases (negative biopsies after recall and not-recalled cases verified by an expert panel). At the end of each session, the radiologists will complete a short questionnaire on the user-friendliness of each AI system. This subjective evaluation will be used in case the objective criteria of detection performance and reading time result in more than one computer system within the non-inferiority margin. The final chosen AI system will be the single one used in the evaluation of the interpretation strategies.

**Fig. 2:**
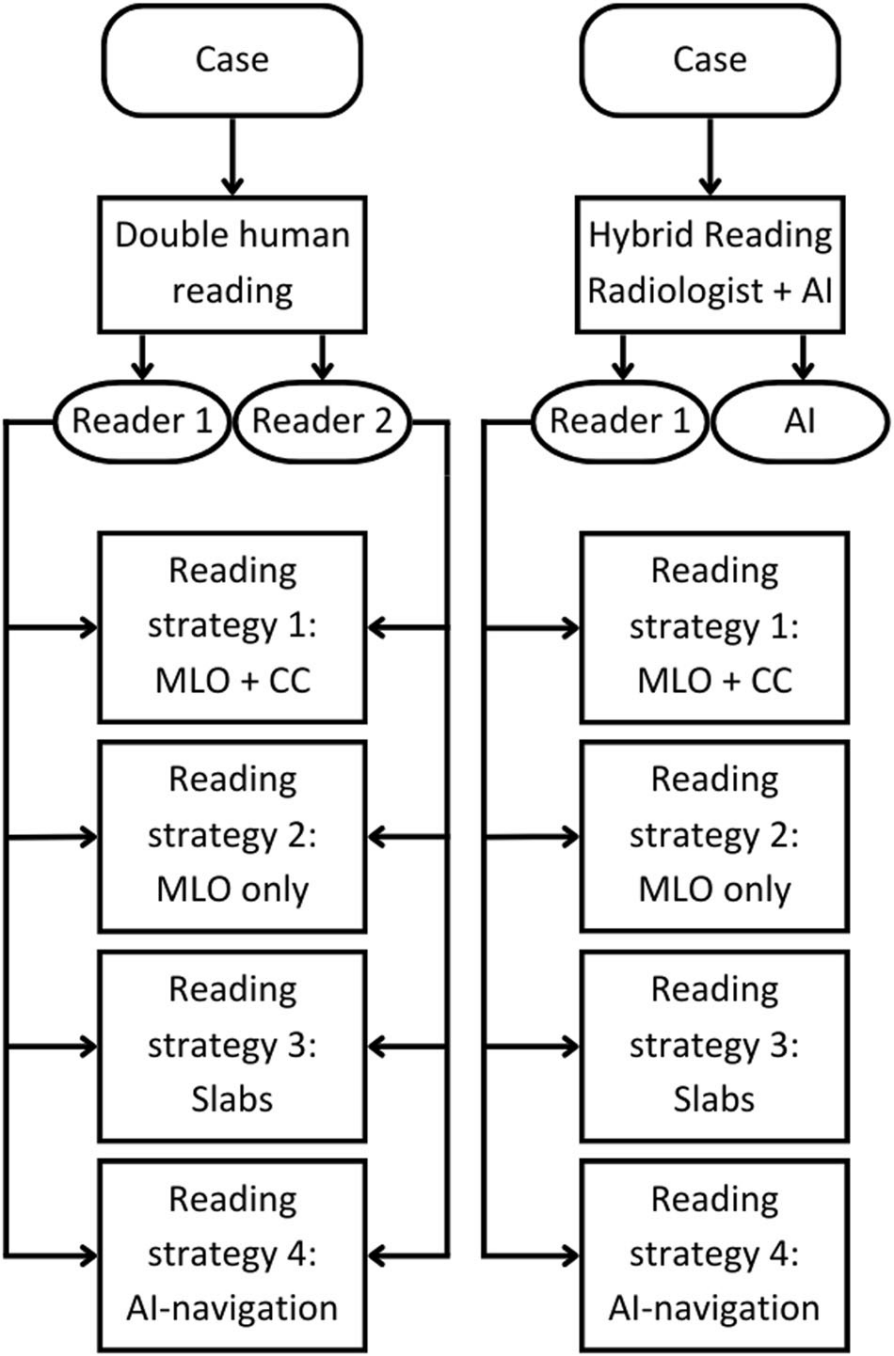
Flowchart of the eight reading strategies evaluated within STREAM. Every screening examination will be evaluated based on two radiologists and one radiologist and one AI prediction. The radiologists will evaluate every screening examination with four different strategies

Four interpretation strategies for individual reading of the cases by a screening radiologist will be evaluated, combined with two different methods to achieve the double-reading of all cases, for a total of eight approaches for interpretation (Fig. 2), as follows:

1. Standard: Acquisition of four views (CC and MLO) of DBT + the corresponding four SM images (Hologic). Interpretation using all images, with the DBT images displayed as stacks of slices with 1 mm separation.
2. Single view DBT (MLO) + SM (MLO): Same as #1 but with interpretation of only the MLO views.
3. Slabbed DBT + SM (CC + MLO for both): Same acquisition as #1 but with the DBT images displayed as 6 mm thick slabs with an overlap of 3 mm (3DQuorum Smart Slices, Hologic).
4. AI-aided navigation: Same as #1 but interpretation performed with an accompanying computer system for navigation and decision support.

The two double-reading strategies will include standard double-reading with a third reader in case of discrepancy between the first two readers, and AI as one reader and a radiologist as the second reader and another radiologist as arbiter in case of discrepancies.

Twenty radiologists will evaluate 200 examinations of the first round with each of the different reading strategies. The 200 examinations will be different from the examinations used for the first reader study, where we compare reading times and user-friendliness. Cases and reading strategies will be randomised in the usual manner, with the necessary four-week wash-out period between sessions. For each interpretation, we will record the location and probability of malignancy score for each suspicious lesion, and a recall/no recall decision, in addition to reading time. We will estimate the JAFROC figure of merit [20], and the case-based AUC for each of the DBT reading strategies studied.

To compare the result of using the standard double-reading by two screening radiologists to that of using hybrid AI-human double-reading, the results of the radiologists reading mentioned above and of the computer system analysis of these 200 cases will be used. For this, first, all interpretations by one radiologist will be paired at random with those of another, to replicate blinded double-reading by two independent radiologists. The AI-based results will be dichotomised into recall/no recall using the operating point that would result in the same average recall rate as the screening radiologists in the first screening round of this trial. Sensitivity and specificity of the final screening outcomes will be the endpoint of these studies.

Once the best reading strategy has been found, all DBT examinations of the second round will be re-interpreted using it. The decision of the first evaluation by the two radiologists will be the gold standard. Wherever possible, different radiologists than the ones who did the first evaluation of the second-round reading will read the corresponding case. The readers will be informed if the second reader is another radiologist or a computer system. The recall rate, breast cancer detection rate, PPV of recall, and interpretation time will be compared to the first evaluation of the second round, allowing for a real-world evaluation in actual screening practice.

### Microsimulation modelling

The results from the prospective trial and reading strategies analyses described above will be used to inform the Microsimulation Screening Analysis (MISCAN)-Breast model to simulate breast cancer screening using DBT. The MISCAN-Breast model is a validated microsimulation model that has been used to estimate the effects of various breast cancer screening scenarios [9, 21–25]. Data on the performance of DBT screening in terms of recall, breast cancer detection, and IC rates will be used to update the model to simulate DBT screening. In addition, the best reading strategy will be simulated. The simulations will be used to predict the long-term effects of DBT screening in the Netherlands in terms of the number of screens performed, breast cancer mortality, life years gained, quality-adjusted life years, and overdiagnosis compared to DM screening and a scenario without screening [9, 26]. The number of overdiagnosed cancers will be estimated by subtracting the number of cancers diagnosed in the scenario without screening from the breast cancers diagnosed in the scenario with screening [27]. In addition, cost-effectiveness will be analysed, in which costs will be based on the best reading strategy.

### Incidental findings

In case a suspicion of breast cancer is identified during the reader study sessions in a participant who was not recalled for work-up, the study radiologists will consult with the coordinating radiologist from the reading unit to decide if recall of this woman is indicated. This protocol is comparable to the protocol used by BVO-NL in case a meeting of screening radiographers results in a suspicion of breast cancer in a participant not recalled.

## Results

Inclusion of participants was started in July 2023 and completed in May 2024. The first trial results are expected to be available late 2024.

## Discussion

The major strength of STREAM is its prospective parallel-group design and its real-world evaluation of different reading strategies. To the best of our knowledge, no prospective trial has evaluated different reading methods for DBT on such a large cohort. This design will give insights into the implementation and acceptability of DBT in the population screening practice.

There are a few limitations to this trial. First, the reader study sessions will start after the first round of data is collected. At that moment, we will not yet have information on ICs. Therefore, the AI solutions will be evaluated on recall and breast cancer detection data from round 1 only. We will re-evaluate the AI solutions once round 2 is completed, but we cannot redo the reading strategy analysis in the funding period. The second evaluation will, therefore, only serve to check if there are significant differences in the results of the AI predictions. This could mean that if a reading strategy including AI is selected as optimal, that strategy does not consider the fact that the AI could be detecting ICs that have not been detected yet. This in turn might influence the evaluation of normal reading versus optimal reading strategy.

Furthermore, in the first round of this trial, there are no previous DBT images available to compare against the current case. Instead, the radiologists are provided with the previous mammogram. Therefore, the first round does not really reflect the possible future steady state in which all priors would consist of DBT images. The second round will have previous DBT images available and will reflect the future steady state more accurately.

The results from the STREAM trial will indicate whether it is beneficial to implement DBT screening in the Netherlands by looking at short-, medium-, and long-term effects, optimal reading strategies, cost-effectiveness, and the acceptability of various stakeholders. In addition, these results will be relevant for other countries considering implementing DBT in screening.

## Supplementary information

ELECTRONIC SUPPLEMENTARY MATERIAL

## Abbreviations

AC: Advanced cancer
AUC: Area under the curve
BVO-NL: Dutch Cancer Screening Organisation (Bevolkingsonderzoek Nederland)
CC: Craniocaudal
DBT: Digital breast tomosynthesis
DM: Digital mammography
IC: Interval cancer
LRCB: Dutch Expert Centre for Screening
MISCAN: Microsimulation Screening Analysis
MLO: Mediolateral-oblique
SM: Synthetic mammography
STREAM: Screening Tomosynthesis trial with advanced REAding Methods
