# Supplementary material for: The Screening Tomosynthesis Trial with Advanced Reader Methods (STREAM): design and rationale of a population-based breast cancer screening trial
Source: Eur Radiol. 2025 Jan 9;35(7):3979–86. doi: 10.1007/s00330-024-11324-z (PMC12165985; doi:10.1007/s00330-024-11324-z)
Supplement: Supplementary file 1 — ELECTRONIC SUPPLEMENTARY MATERIAL [file 330_2024_11324_MOESM1_ESM.pdf]

The Screening Tomosynthesis Trial with Advanced Reader Methods (STREAM): Design and Rationale of a Population-Based Breast Cancer Screening Trial

ELECTRONIC SUPPLEMENTARY MATERIAL

Appendix A – Questionnaire participants

Why this questionnaire?

Thank you for participating in our study. You have made breast images in a different way (tomosyntheses). We would like to know how you experienced this. Therefore, we want to ask you to complete this questionnaire. Completing the questionnaire will not take long (5 to 10 minutes).

In the images below, you can see again what the difference is between the two ways of making breast images.

Mammography

At the moment, mammography is used in the screening programme. It is used to make two images of each breast. Mammography is a good method to detect breast cancer.

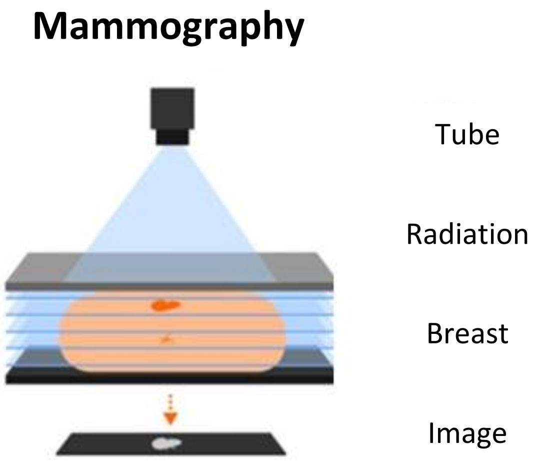

Tomosynthesis

Tomosynthesis is a different way to detect breast cancer. A tomosynthesis is done with the same machine as a mammogram. The only difference is that the tube above the breast will move to make multiple images.

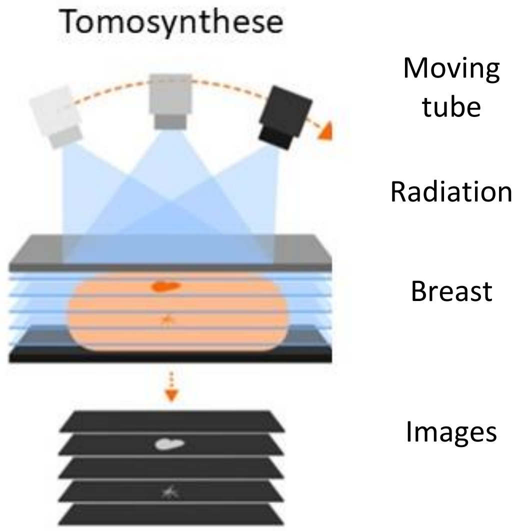

Experience with tomosynthese

1. In which screening centre did you participate in the breast cancer screening programme?
    a. Groningen
    b. Deventer
    c. Apeldoorn
    d. A mobile unit near Den Bosch
    e. A mobile unit near Arnhem – Zutphen
    f. A mobile unit near Huizen (Noord-Holland)

2. Below are some experiences mentioned that you may have had during the tomosyntheses exam. Check for each experience the box that comes closest to your experiences. Check one box per question.

Did you experience the following during your tomosynthese exam?

		No, not at all	Yes, a bit	Yes, quite some	Yes, a lot
2.1.	I experienced the tomosynthesis exam as painful.	☐	☐	☐	☐

		No, not at all	Yes, a bit	Yes, quite some	Yes, a lot
2.2.	I experienced the tomosynthesis exam as uncomfortable.	☐	☐	☐	☐

		No, not at all	Yes, a bit	Yes, quite some	Yes, a lot
2.3.	I was scared during the tomosyntheis exam	☐	☐	☐	☐

3. How much pain did you experience while making the breast images with tomosynthesis?

   Circle the number on the scale for your pain, in which 0 stands for ‘no pain’ and 10 for the ‘worst pain thinkable’.

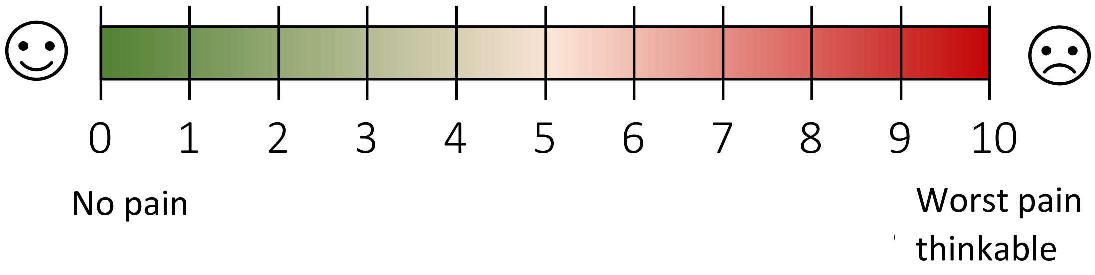

In the STREAM study, we use tomosynthesis for breast imaging during two rounds of the screening programme. This means that you will be invited for another tomosynthesis in about two years.

4. Are you planning to have tomosynthesis images made during the next round of the screening programme, instead of mammography?
   a. Yes [go to question 7]
   b. No [go to question 5]
   c. I don’t know [go to question 6]

5. Why wouldn’t you have another round of tomosynthesis imaging?

[open question] [go to question 7]

6. Why don’t you know yet if you would have another round of tomosynthesis imaging?

   [open question]

Comparison to mammography

7. Have you participated in previous rounds of the screening programme in which mammography was used?
   a. Yes [go to question 8]
   b. No, I did not participate after previous invitations [go to question 11]
   c. No, I only now received by first invitation [go to question 11]
   d. Prefer not to say [go to question 11]

8. How much pain did you experience while making the breast images with mammography?

When comparing to the score you assigned to tomosynthesis imaging, how would you score mammography? [SYSTEM COPIES TOMOSYNTHESIS SCORE} Circle the number on the scale for your pain, in which 0 stands for ‘no pain’ and 10 for the ‘worst pain thinkable’.

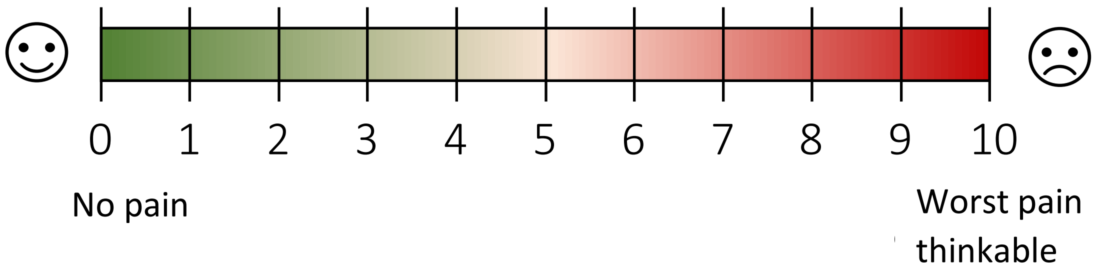

9. Which method of breast imaging do you prefer?
   a. Mammography
   b. Tomosynthesis
   c. No preference

10. Why do you prefer this method?

Next examination

11. If you would participate in the next round of the screening programme, does it matter which imaging method is used?

Would you participate if you were invited for a mammogram?	Yes ☐	No ☐
Would you participate if you were invited for a tomosynthese?	Yes ☐	No ☐

12. In the next round of the screening programme, you will receive an invitation to have imaging with tomosynthesis. Do you have ideas to improve this second round of the study?

[open question]

General questions

13. In which year were you born?

[drop down with calendar years]

14. What is your highest completed education? (That means, a study completed with a diploma or certificate)
    a. Primary school
    b. Vmbo, or first three years of havo/vwo
    c. MBO, level 1
    d. MBO level 2, 3, or 4, or MBO before 1998
    e. Havo or vwo (completely)
    f. HBO bachelor or WO bachelor
    g. HBO master or WO master or doctorate
    h. Other, …
    i. Prefer not to disclose

15. Do you want to mention anything else about you experience with tomosynthesis or this study?

[open question]

Thank you for completing this questionnaire!

U are helping us to improve the breast cancer screening programme.

The study-team

STREAM

Appendix B – Questionnaire employees

Radiographers

Lately, you have been cooperating on the first round of the STREAM study in which tomosynthesis was used in the breast cancer screening programme. We would like to know how you experience working with tomosynthesis and if you have any suggestions for improvements. Therefore, we ask you to complete a short questionnaire.

1. What was your experience with tomosynthesis?
    a. Good
    b. Workable, but not optimal
    c. Difficult to use

[open field provided for explanation]

2. After some time of getting used to it, how long did it take to use tomosynthesis compared to mammography?
    a. Just as long as mammography
    b. 1-25% longer
    c. 26-50% longer
    d. 51-75% longer
    e. 76-100% longer
    f. >100% longer

[open field for explanation]

3. Did you experience problems while using tomosynthesis with the following: (multiple answers are possible)
    a. Positioning of the breast
    b. The period of standing still
    c. The use of the compression paddle
    d. Giving compression
    e. The use of the mammographer
    f. The use of the acquisition station
    g. The use of the working station
    h. The signaling
    i. Other, … [open field]
    j. No problems

3.1. [for each of the problems experienced] What was the problem with [problem]?

[open question]

4. Do you think tomosynthesis is workable in the screening setting?

[open question]

5. Which technique do you prefer to work with in screening?
    a. Mammography
    b. Tomosynthesis
    c. No preference

6. Why do you prefer this technique?

    [open question]

7. Are you satisfied with the training you received on tomosynthesis before the study started?
    a. Yes [go to question 9]
    b. No [go to question 8]

8. Which information did you miss in the training?

    [open question]

9. Do you have the impression that clients were sufficiently informed about the study upon arrival at the screening unit?
    a. Yes [go to question 12]
    b. No [go to question 10]
    c. I don't know [go to question 10]

10. Which information could be altered/improved in the invitation letter?

    [open question]

11. Which information could be altered/improved in the study leaflet?

    [open question]

12. Do you have the impression that there were aspects that were determined for clients to participate or not?
    a. Yes [go to question 13]
    b. No [go to question 15]

13. Which aspects were determined for clients to participate?

[open question]

14. Which aspects were determined for clients to not participate?

[open question]

15. Do you have suggestions for improvements for the second round?

[open question]

16. How long are you working as a radiographer in a screening setting?

[open field]

17. Do you have anything else that you want to let us know about your experience with tomosynthesis or this study?

[open field]

Thank you for completing this questionnaire!

Radiologists

Lately, you have been cooperating on the first round of the STREAM study in which tomosynthesis was used in the breast cancer screening programme. We would like to know how you experience working with tomosynthesis and if you have any suggestions for improvements. Therefore, we ask you to complete a short questionnaire.

Unfortunately, we experienced some issues in loading speed during the first months on the STREAM study. With this questionnaire we want to investigate your experience with tomosynthesis in screening during a ‘steady-state’ situation. Therefore, we ask you to answer the questions on your experience during the period between December 2023 and now.

1. Did you read tomosynthesis images before? For example for diagnostic purposes in the hospital?
    a. Yes
    b. No

2. What is your experience with the scoring of tomosynthesis images in screening compared to mammography?
    a. Good
    b. Workable, but not optimal
    c. Difficult

[open field for explanation]

3. After some time of getting used to it, how long did it take to read and score tomosynthesis images compared to mammography?
    a. Just as long as mammography
    b. 1-25% longer
    c. 26-50% longer
    d. 51-75% longer
    e. 76-100% longer
    f. >100% longer

[open field for explanation]

4. Did you experience difficulties with the reading and scoring of tomosynthesis images regarding:
   (multiple answers possible)
    a. Worse positioning of the mamma
    b. Lower quality/ motion blur
    c. The use of the workstation
    d. The use of the hanging protocol
    e. The signaling of radiographers
    f. The comments from radiographers
    g. The use of the BI-RADS classification
    h. Other, …
    i. No problems

4.1 [for each of the problems experienced] What was the problem with [problem]?

[open question]

5. To your experience, did you score more or less BI-RADS 0 classifications with tomosynthesis compared to mammography?
    a. Less BI-RADS 0 compared to mammography
    b. Equal amount of BI-RADS 0 as with mammography
    c. More BI-RADS 0 compared to mammography
    d. I don’t know

6. To your experience, did you see calcifications better or worse with tomosynthesis compared to mammography?
    a. Calcifications were better visible compared to mammography
    b. Calcifications were equally visible as with mammography
    c. Calcifications were less visible compared to mammography
    d. I don’t know

7. How did you experience the comparison of tomosynthesis images to previous mammography images?
    a. Good
    b. Workable, but not optimal
    c. Hard to compare

[open field for explanation]

8. Which technique do you prefer to work with in screening?
    a. Mammography
    b. Tomosynthesis
    c. No preference

9. Why do you prefer this technique/ Why do you have no preference?

[open question]

10. Are you satisfied with the training you received on tomosynthesis before the study started?
    a. Yes [go to question 12]
    b. No [go to question 11]

11. Which information did you miss in the training?

[open question]

12. In September and October we implemented improvements to the loading speed of tomosynthesis images. Are you satisfied with these improvements?
    a. Yes [go to question 13]
    b. No [go to question 14]

13. Why are you satisfied?

[open question] [go to question 15]

14. Why aren't you satisfied?

[open question]

15. Do you have suggestions for improvements for the second round?

[open question]

16. Do you have anything else that you want to let us know about your experience with tomosynthesis or this study?

[open field]

Thank you for completing this questionnaire!

Appendix C - Sample size calculation

The sample size estimate for this study was performed using G*Power (version 3.1.9.4). We chose the following input parameters: alpha = 0.05, beta = 0.20 (power = 80%), two-sided Z-test, and allocation ratio for the control/intervention group = 5. Baseline data on screening outcomes in the Dutch programme with DM are:

- interval cancer (IC) rate DM = 2.2 / 1000 [1,2], and
- advanced cancer rate (AC, stage II+) DM = 1.38 / 1000 [3].

Based on the available evidence so far, the expected difference in interval cancer rate varies from −0.15 / 1000 to −1.20 / 1000 [4-6]. To the best of our knowledge, only one study so far, the Verona pilot study, has reported data on the AC rate [7]. That study showed that at repeat screening, the proportion of cancers stage II or higher was 14.5% (19 of 131 cancers) with DBT+SM and 8.5% (five of 59 cancers) with DBT+DM, both of which were lower than the proportion in the control group with DM only (30 of 110 cancers, 27.3%) ($P \leq 0.01$).

We powered our study to detect a reduction in the combined endpoint of IC and AC rate of 1.3 / 1000, which reflects a difference of 36% from this combined endpoint with DM (3.6 / 1000). This requires a sample size for the intervention group in the second round of 17,275 screening examinations with DBT. In turn, this requires 18,184 screening examinations with DBT in the first round, assuming 95% re-attendance. The sample size for the control group is five times that of the intervention group in the second round, i.e., 86,375 women screened with DM. For practical reasons, the sample sizes were rounded up to 18,200 in the intervention group and to 91,000 in the control group.

Based on a screening participation rate of 75% and an expected study participation rate of 60%, we will need to invite about 40,400 women to acquire 18,200 DBT exams. A screening unit performs 14,000 examinations, on average, per year. To ensure an adequate distribution across the Dutch population and perform the study in an acceptable time period, we will involve six screening units.

References Appendix

1. Integraal Kankercentrum Nederland (IKNL) (2020) Monitor van het bevolkingsonderzoek borstkanker 2018-2019. Nijmegen: Integraal Kankercentrum Nederland (IKNL): www.iknl.nl/borstkankermonitor.

2. Sankatsing VDV, Fracheboud J, de Munck L, et al (2018) Detection and interval cancer rates during the transition from screen-film to digital mammography in population-based screening. BMC Cancer: 18(1):256. doi: 10.1186/s12885-018-4122-2.

3. de Munck L, Siesling S, Fracheboud J, den Heeten GJ, Broeders MJM, de Bock GH (2020) Impact of mammographic screening and advanced cancer definition on the percentage of advanced-stage cancers in a steady-state breast screening programme in the Netherlands. Br J Cancer: 123(7):1191–1197. doi: 10.1038/s41416-020-0968-6.

4. Houssami N, Zackrisson S, Blazek K, et al (2021) Meta-analysis of prospective studies evaluating breast cancer detection and interval cancer rates for digital breast tomosynthesis versus mammography population screening. Eur J Cancer: 148:14–23. doi: 10.1016/j.ejca.2021.01.035.

5. Johnson K, Lång K, Ikeda DM, Åkesson A, Andersson I, Zackrisson S. (2021) Interval Breast Cancer Rates and Tumor Characteristics in the Prospective Population-based Malmö Breast Tomosynthesis Screening Trial. Radiology: 204106. doi: 10.1148/radiol.2021204106.

6. Pulido Carmona C. Interval breast cancer in a screening programme: comparison between women screened with two-dimensional digital mammography or tomosynthesis. European Congress of Radiology. Vienna, Austria (virtual)

7. Caumo F, Montemezzi S, Romanucci G, et al (2021) Repeat Screening Outcomes with Digital Breast Tomosynthesis Plus Synthetic Mammography for Breast Cancer Detection: Results from the Prospective Verona Pilot Study. Radiology, 298(1): p. 49-57.
